# Effects of Hot Stamping and Tempering on Hydrogen Embrittlement of a Low-Carbon Boron-Alloyed Steel

**DOI:** 10.3390/ma11122507

**Published:** 2018-12-10

**Authors:** Yongjian Zhang, Weijun Hui, Xiaoli Zhao, Cunyu Wang, Han Dong

**Affiliations:** 1 School of Mechanical, Electronic and Control Engineering, Beijing Jiaotong University, Beijing 100044, China; wjhui@bjtu.edu.cn (W.H.); zhaoxiaoli@bjtu.edu.cn (X.Z.); 2 Center Iron and Steel Research Institute, Beijing 100081, China; wang_cunyu@126.com; 3 School of Materials Science and Engineering, Shanghai University, Shanghai 200444, China; 13910077790@163.com

**Keywords:** boron-alloyed steel, hot stamping, hydrogen embrittlement, tempering, microstructure

## Abstract

The effects of hot stamping (HS) and tempering on the hydrogen embrittlement (HE) behavior of a low-carbon boron-alloyed steel were studied by using slow strain rate tensile (SSRT) tests on notched sheet specimens. It was found that an additional significant hydrogen desorption peak at round 65–80 °C appeared after hydrogen-charging, the corresponding hydrogen concentration (*C*_Hr_) of the HS specimen was higher than that of the directed quenched (DQ) specimen, and subsequent low-temperature tempering gave rise to a decrease of *C*_Hr_. The DQ specimen exhibited a comparatively high HE susceptibility, while tempering treatment at 100 °C could notably alleviate it by a relative decrease of ~24% at no expanse of strength and ductility. The HS specimen demonstrated much lower HE susceptibility compared with the DQ specimen, and tempering at 200 °C could further alleviate its HE susceptibility. SEM analysis of fractured SSRT surfaces revealed that the DQ specimen showed a mixed transgranular-intergranular fracture, while the HS and low-temperature tempered specimens exhibited a predominant quasi-cleavage transgranular fracture. Based on the obtained results, we propose that a modified HS process coupled with low-temperature tempering treatment is a promising and feasible approach to ensure a low HE susceptibility for high-strength automobile parts made of this type of steel.

## 1. Introduction

One of the most important challenges for the automobile industry is to further enhance passenger safety coupled with a simultaneous reduction of vehicle weight regarding energy consumption and exhaust emissions [1]. This has notably promoted the research and development of ultra-high strength steels (UHSSs) in the modern automobile industry. However, the application of these steels usually leads also to some disadvantages such as unacceptable high stresses during conventional cold forming and significant spring-back [2,3,4]. In spite of the great efforts that have been conducted to overcome these problems, cold press forming of complicated shape parts is still difficult when the strength level of a steel sheet is as high as ~1500 MPa [4,5]. Therefore, new forming technologies called hot stamping (HS), hot forming, and press hardening or die quenching have been developed for manufacturing automobile structural parts with a strength level of ~1500 MPa or more in recent years [2,3,4,5,6,7,8,9,10].

HS is an innovative non-isothermal high-temperature forming process, in which complicated shape parts with an ultra-high strength level are produced, primarily for the purpose of eliminating spring-back [3,4]. During the process, the blanks are heated to the austenitizing temperature in a furnace to form a homogeneous austenitic microstructure, and then immediately transferred to a press where they are simultaneously formed and quenched at a controlled cooling rate between continuously internally cooled dies to ensure the austenitic microstructure transforms into a martensitic one. Such HS automobile components are mostly structural elements like A/B-pillar (Pillars are the vertical or near vertical supports of a car’s window area—designated respectively as the A, B, C or (in larger cars) D-pillar, moving from the front to rear), bumper, tunnel and rocker and roof rail [6]. The applications of HS technology in the global automotive industry are expanding rapidly. However, it is well known that, in general, untempered martensitic microstructure is most susceptible to hydrogen embrittlement (HE). The HE susceptibility increases with an increase in steel strength level, especially when it is higher than ~1000–1200 MPa [11,12,13]. HE can be induced for low strength steel under massive hydrogen penetration, while very little amounts of hydrogen can be catastrophic in the case of high strength steel. Therefore, HE has been one of the most frustrating problems restricting the use of UHSSs for parts under loading, especially in hydrogen-rich environments [14,15,16].

Boron-alloyed steels such as 22MnB5, 27MnCrB5 and 37MnB4, which produced a fully martensitic microstructure after HS when water-cooled dies were applied, have been the point of focus for the material selection in HS [3,4,6]. Boron is the element which affects the hardenability the most, and the addition of a small amount of boron could guarantee obtaining nearly fully martensitic microstructure over the cross-section of the component after water cooling [4,6]. Among these boron-alloyed steel grades, 22MnB5 is the most widely used steel grade in the HS practice, although other boron-alloyed steels, such as 22MnMoB [17] and even non-boron-alloyed steels [3] and medium-Mn steel [9,18], were also explored. Most of the research activities carried out in the framework of HS are aimed at improving the efficiency of HS process and numerical simulating it. More recently, a significant portion of the research is focused on HS of boron-alloyed steels with tailored properties, i.e., components with a combination of strong and soft properties via obtaining different microstructures in one stamped component [6,17,19].

The detrimental effects of hydrogen on the performance of key components and structures made of high strength steels are well known and have been studied extensively. However, there are only a few published data available concerning the HE susceptibility of hot stamped steels [14,20,21,22,23,24]. This research was focused on the effect of microalloying elements Nb [20,21] and Ti [22] on the HE susceptibility of HS boron-alloyed steels, the absorption and desorption of diffusible hydrogen in Al-Si coated boron-alloyed steel [23] and the effect of tempering on the HE of 22MnB5 steel [24]. Unfortunately, few studies were paid to the HE behavior of boron-alloyed steels after HS [14], especially to the combined effect of HS and tempering. Therefore, in the present investigation, the influences of HS and low-temperature tempering on the HE behavior of a low-carbon boron-alloyed steel were studied by using slow strain rate tensile (SSRT) tests, in an attempt to ensure the safety performance of automobile parts made of this type of steel grade under hydrogen-related service environments.

## 2. Material and Experimental Procedures

**Figure 1:**
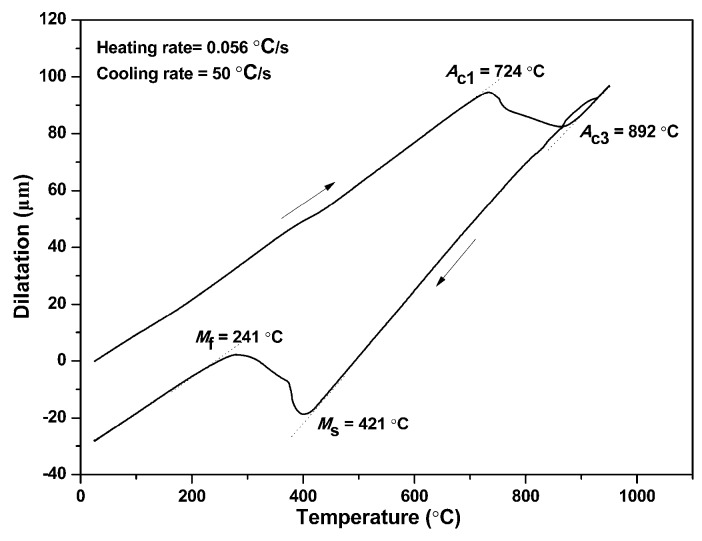
Dilatometric curve of the tested steel austenitized at 940 °C for 10 min and cooled at 50 °C/s, showing ascertaining the start and finish temperatures of phase transformation by tangent method.

**Figure 2:**
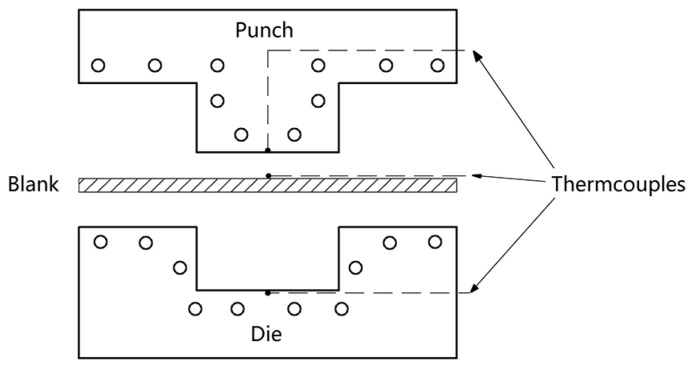
Schematic illustration of the designed tool for hot stamping experiment.

**Figure 3:**
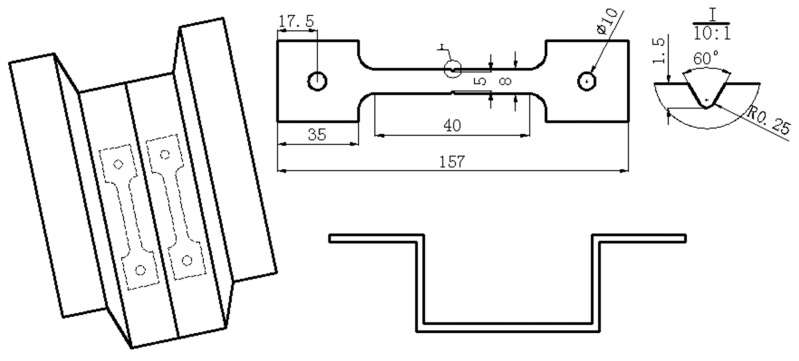
Schematic of tensile test specimens cutting from the hot stamped blank (unit: mm).

The material used is a novel low-carbon boron-alloyed steel with a nominal composition of 0.20C-0.85Si-1.60Mn-0.006P-0.002S-0.0015B (wt.%), and was supplied in the form of 1.5-mm-thick cold-rolled sheets. The steel sheets were produced through converter smelting following ladle furnace refining, continuous casting and continuous rolling. The critical temperatures of the tested steel were obtained by means of dilatometric measurement using 1.5 mm × 4 mm × 10 mm specimens on a Bähr DIL805L quenching device (Bähr-Thermoanalyse GmbH, Hüllhorst, Germany) as shown in Figure 1. Samples of 200 mm-width and 300 mm-length were cut from the 1.5-mm-thick cold-rolled sheets. The samples were austenitized at 940 °C for 5 min in a nitrogen-protected electric-resistance furnace (ECOO Co., Ltd., Yancheng, China) and then quickly moved to a designed HS device with continuously internally cooled water as shown in Figure 2, and finally hot stamped. For comparison, part of the samples was directly water quenched, i.e., without HS (called DQ hereafter). Parts of the DQ and HS samples were tempered at 100 °C and/or 200 °C for 120 min and air cooled (called DQ-T100 and HS-T200 specimens, respectively). The selection of different tempering temperatures is mainly for the purpose of studying the effectiveness of the modified HS schedule as well as the influence of low-temperature tempering treatment on alleviating the susceptibility to HE. The cutting of tensile sheet specimens is shown in Figure 3. Smooth tensile sheet specimens for tensile testing were cut to a 1.5 mm thickness, 8 mm width and 40 mm gauge length. Notched tensile specimens with a notch root radius of 0.25 mm, notch depth of 1.5 mm (stress concentration factor *K*_t_ = 3.8) were used for the SSRT tests.

Hydrogen was introduced into the specimens through an electrochemical method in a 0.1 mol/L NaOH aqueous solution at ambient temperature. The current density was 8 mA/cm^2^ and the charging time was 72 h. After hydrogen-charging, the SSRT specimens were mechanically polished and then electroplated with a Zn coating with a thickness of ~20 μm to prevent hydrogen release using a Zn plating solution recommended by ISO 16573:2015 [25]. The Zn electroplating was started within 5 min after the end of the hydrogen-charging and was conducted at an applied current density of 38 mA/cm^2^ for 5 min at a temperature of 55 °C. To homogenize the hydrogen distribution within the specimens as much as possible, the Zn coated specimens were kept at room temperature for 24 h before the SSRT tests [26].

Tensile tests were conducted on a universal testing machine at a crosshead speed of 1 mm/min, while the SSRT tests were performed on a Bairoe YYF-50 tensile testing machine operated at a constant crosshead speed of 0.005 mm/min at ambient temperature. Depending on the fracture stress of the specimens, the duration of the SSRT tests ranged from 4 h to 5 h. After the SSRT tests, the notch tensile strengths (*σ*_N0_ and *σ*_NH_ for the uncharged and hydrogen-charged specimens, respectively), which were defined as the nominal maximum tensile stresses, were obtained. The relative notch tensile strength loss was defined as an index to evaluate the relative susceptibility to HE (HEI):

(1) $$HEI(\%)=(1-\frac{\sigma_{NH}}{\sigma_{N0}})\times 100\%$$

Scanning electron microscopy (SEM, Zeiss EVO18, Carl Zeiss Microscopy Ltd., Cambridge, UK) was used to observe the SSRT facture surfaces. The analysis of hydrogen was conducted on a thermal desorption spectrometry (TDS, HTDS-002, R-DEC Co., Ltd., Tsukuba, Japan). The specimen was heated from room temperature to 800 °C at a constant heating rate of 100 °C/h, and a quadrupolar mass spectrometer (R-DEC Co., Ltd., Tsukuba, Japan) was used to analyze the hydrogen effusing out of the specimen during heating.

Optical microscopy (OP, Carl Zeiss Microscopy GmbH, Göttingen, Germany), SEM (Carl Zeiss Microscopy Ltd., Cambridge, UK) and transmission electron microscopy (TEM, FEI, Hillsboro, OR, USA) were used for microstructural characterization. The specimens for SEM and OP were etched in a 3% nital solution after standard grinding and polishing. The foils for TEM were electro-polished in a twin-jet electro-polishing apparatus using an alcohol solution with 5 vol.% perchloric acid at −20 °C and were then examined in a TEM at an accelerating voltage of 200 kV. The amount of precipitation phase of the tested specimens was obtained through physical-chemical phase analysis [27].

Auger electron spectroscopy (AES, ULVAC-PHI, Inc., Chigasaki, Japan) was applied for electron backscatter diffraction (EBSD) measurements utilizing a PHI 710 microprobe operating (ULVAC-PHI, Inc., Chigasaki, Japan) at 20 kV with a step size of 0.04 μm. Samples for the EBSD examination were electro-polished in the same solution as that for the TEM foils. The dislocation density of the specimens was determined by analyzing the *X*-ray diffraction (XRD) patterns via the Rietveld software, Materials Analysis Using Diffraction (MAUD, 2.071). The XRD measurements were conducted between scanning rage of 40° to 100°, rate of 2°/min and step size of 0.05° at room temperature using a Cu-K_α_ radiation source. For more details about the experimental procedure see Ref. [28].

## 3. Results

### 3.1. Microstructure Characteristics

**Figure 4:**
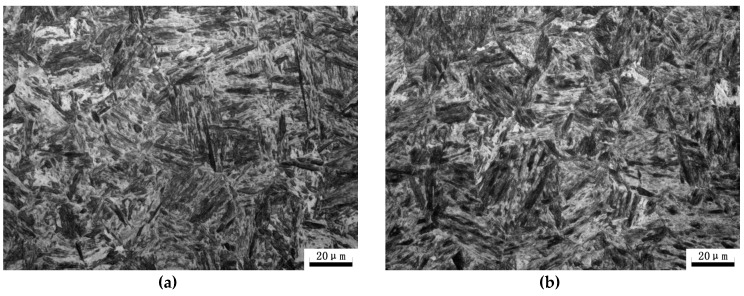
Optical microstructures of the (**a**) DQ and (**b**) HS specimens.

**Figure 5:**
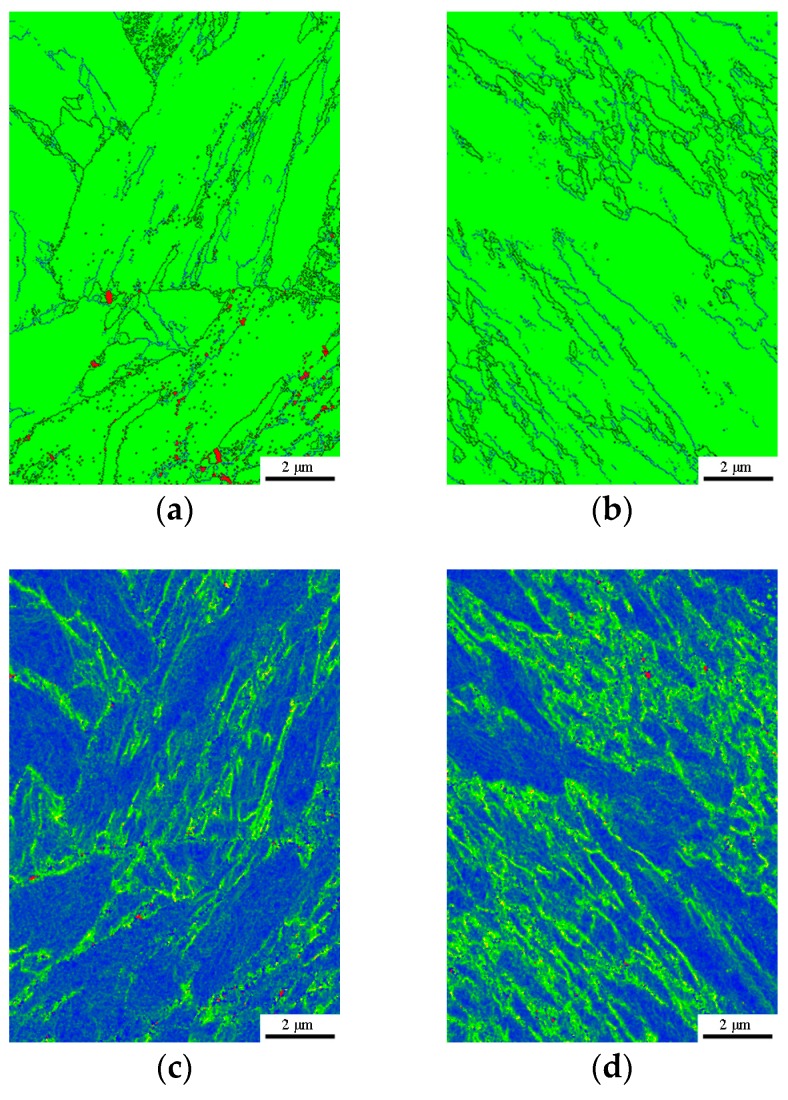
(**a**,**b**) EBSD phase maps of the DQ and HS specimens, green color refers to BCC and red color refers to FCC, the black lines are high-angle boundaries with misorientation angles over 15° while the blue lines are low-angle boundaries with misorientation angles between 2° and 15°; (**c**,**d**) EBSD-KAM of the DQ and HS specimens. (**a**,**c**) DQ; (**b**,**d**) HS.

**Figure 6:**
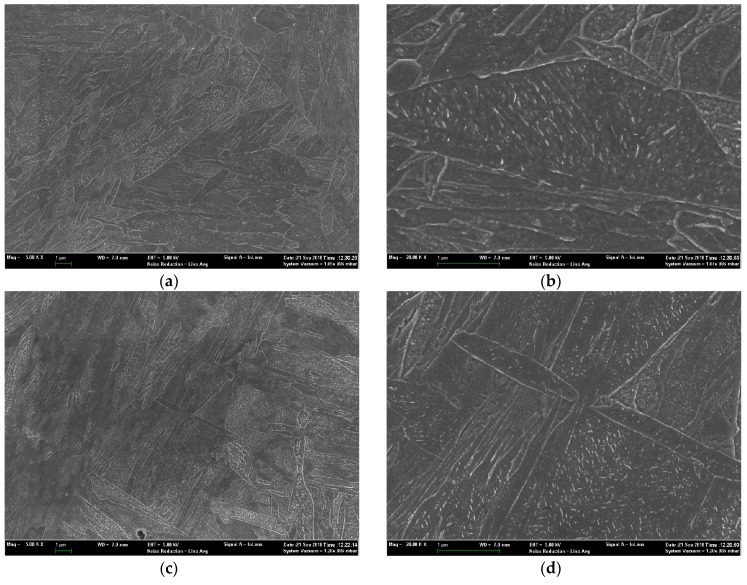
SEM micrographs of the (**a**,**b**) DQ and (**c**,**d**) HS specimens, showing the morphology and distribution of fine carbides.

**Figure 7:**
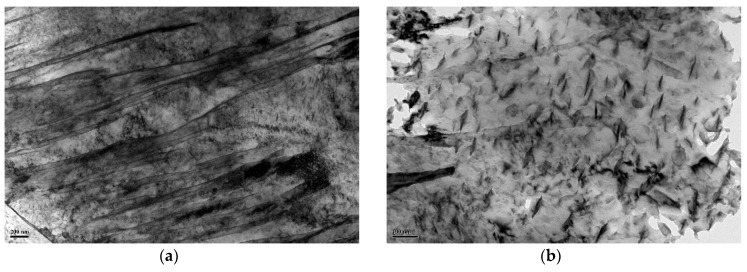

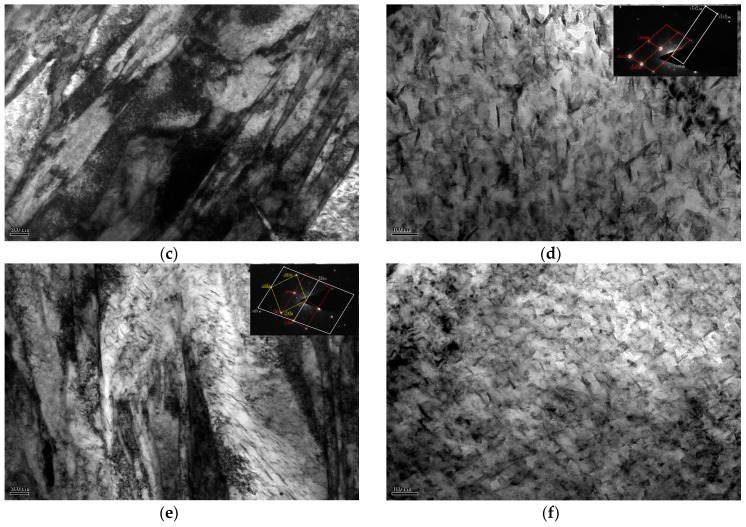
TEM micrographs of the (**a**,**b**) DQ, (**c**,**d**,**e**) HS and (**f**) DQ-T100 specimens, showing martensitic laths and the precipitation of fine and needle-shaped *ε*- and/or *θ*-carbides (insert: selected area electron diffraction patterns of the particles in (**d**) and (**e**)).

As expected, both the DQ and HS specimens exhibit a full lathy martensitic microstructure as shown in Figure 4 and Figure 5, which revealed that the designed HS schedule is reasonable. Figure 6 presents the SEM micrographs of the DQ and HS specimens. All the SEM micrographs exhibit lathy martensitic structures with abundant fine and need-shaped carbides within laths as well as along lath boundaries. Further TEM observations revealed that these carbides are *ε*- and/or *θ*-carbides and were primarily precipitated within martensitic laths (Figure 7). This reveals that these carbides were already precipitated upon either the conventional quenching or HS processes. Detailed analysis of the mass fraction of carbides is present in Table 1. The DQ and DQ-T100 specimens show an almost identical carbide fraction, while the HS specimen exhibits a higher carbide fraction. Moreover, the HS specimen has higher dislocation density than the DQ specimen, as can also be seen from Figure 7, while tempering at 200 °C of the HS specimen (the HS-T200 specimen) caused a decrease of dislocation density. The kernel average misorientation (KAM) value, which is regarded to be proportional to the micro-strain induced by crystal defects such as dislocations [29], was measured using the SEM EBSD phase maps shown in Figure 5a,b. The average KAM values of the DQ and HS specimens are ~0.64° and ~0.54°, respectively, which also indicates that the HS specimen has higher dislocation density than the DQ specimen.

### 3.2. Mechanical Properties

Table 1 presents the mechanical properties of the samples under different conditions. The HS specimen exhibits almost identical ultimate tensile strength (UTS) and total elongation (TEL) to those of the DQ specimen, while the yield strength (YS) of the former is slightly higher than the latter. These results reveal that the HS specimen has comparable mechanical properties to the DQ specimen. Tempering treatment of the DQ specimen at 100 °C and the HS specimen at 200 °C gave rise to an increase of both the YS and TEL, while a slight decrease of the UTS occurred.

### 3.3. Hydrogen Absorption and Desorption Behavior

**Figure 8:**
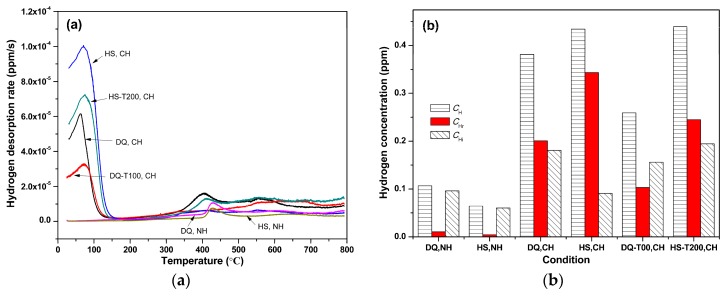
(**a**) Hydrogen desorption curves and (**b**) corresponding hydrogen concentration of the uncharged and hydrogen-charged specimens under different conditions.

The hydrogen desorption rate vs. heating temperature curves of the specimens under different conditions are presented in Figure 8a. The uncharged specimens exhibited a lower height desorption peak at ~430 °C (high-temperature peak), while the hydrogen-charged specimens exhibited an additional higher height desorption peak at round 65–80 °C (low-temperature peak). It was found that the hydrogen corresponding to this low-temperature peak can gradually diffuse out during exposure at room temperature in martensitic steels [27,30], and therefore, it is considered that diffusible or reversible hydrogen leads to a deterioration of mechanical properties [26]. The hydrogen corresponding to this high-temperature peak represents non-diffusible or irreversible hydrogen at room temperature, which is regarded to not lead to a degradation of mechanical properties [31]. Thus, the whole hydrogen concentration (*C*_H_) can be roughly regarded as the sum of reversible hydrogen concentration (*C*_Hr_) and irreversible hydrogen concentration (*C*_Hi_). As shown in Figure 8b, almost all the hydrogen is irreversible hydrogen for the uncharged specimens, while most of the introduced hydrogen corresponds to diffusible hydrogen. The *C*_Hr_ of the HS specimen is larger than that of the DQ specimen while the *C*_Hi_ of the former is lower than that of the latter. Low-temperature tempering treatment caused a decrease of the *C*_Hr_ for both the DQ and HS specimens while it caused an increase of the *C*_Hi_ for the HS specimen and a slight decrease for the DQ specimen. Moreover, the hydrogen desorption peak temperature slightly increased after low-temperature tempering treatment, indicating more stablity of the diffusible hydrogen.

### 3.4. HE Behavior

**Figure 9:**
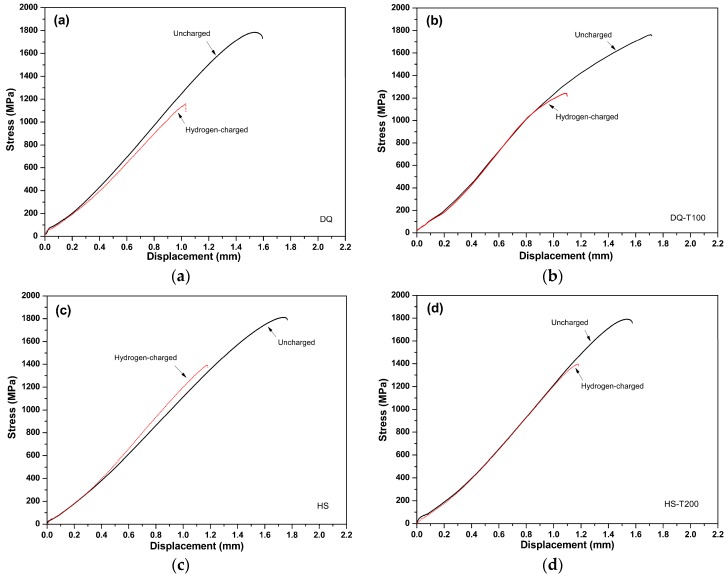
SSRT curves of the notched sheet specimens before and after hydrogen-charging. (**a**) DQ; (**b**) DQ-T100; (**c**) HS; (**d**) HS-T200.

Figure 9 gives the typical SSRT curves obtained for the notched sheet specimens under different conditions. Since the specimens were notched, which is regarded to be appropriate to reflect the susceptibility to HE of high strength steels [26,32], the displacement is short and thus showed an early fracture before distinct yielding and necking, revealing the remarkable effect of notch on the tensile behavior. Hydrogen-charging remarkably deteriorated the tensile behavior, that is, both the maximum notch tensile stress and the fracture displacement were lowered.

The measured notch tensile strengths and the calculated values of HEI are presented in Table 2. It is obvious that all the specimens have nearly identical *σ*_N0_ mainly because they have similar smooth mechanical properties, as shown in Table 1. The *σ*_NH_ increases while the HEI decreases gradually in the following order: DQ, DQ-T100, HS and HS-T200. The DQ specimen has the highest HE susceptibility and tempering treatment at 100 °C could notably alleviate it. Notably, the HS specimen demonstrates much higher *σ*_NH_ than the DQ specimen. The *σ*_NH_ of the HS specimen is also remarkably higher than that of the DQ-T100 specimen, and tempering treatment at 200 °C could further enhance it. These results show that the HS specimen has much lower susceptibility to HE than the DQ specimen does, indicating the superiority of the designed HS schedule.

### 3.5. SSRT Fracture Surface Characteristics

**Figure 10:**
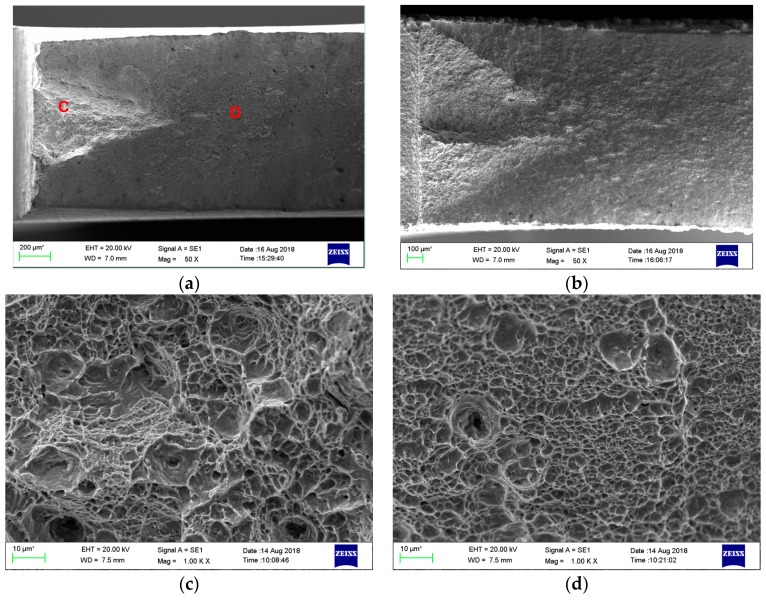
SEM fractographs of the (**a**,**c**,**d**) uncharged and (**b**) hydrogen-charged HS specimens after the SSRT tests, showing a view of (**a**,**b**) low magnification and high magnification of the areas (**c**) C and (**d**) D in (**a**).

A detailed SEM examination of the fracture surfaces was carried out after the SSRT tests. As shown in Figure 10a,b, the low magnification fracture surface morphology is typical of notched specimens with a triangular region near to the notch, which is primarily connected with the stress intensification region [14,24]. Hydrogen-charging remarkably enlarged this area. As for the uncharged specimen, high magnification observation revealed a typical dimple ductile fracture comprising of dimples with different sizes in the triangular area (Figure 10c), while it is also a dimple ductile fracture with fine and uniform dimples, except a few large ones in the crack propagation region (Figure 10d).

**Figure 11:**
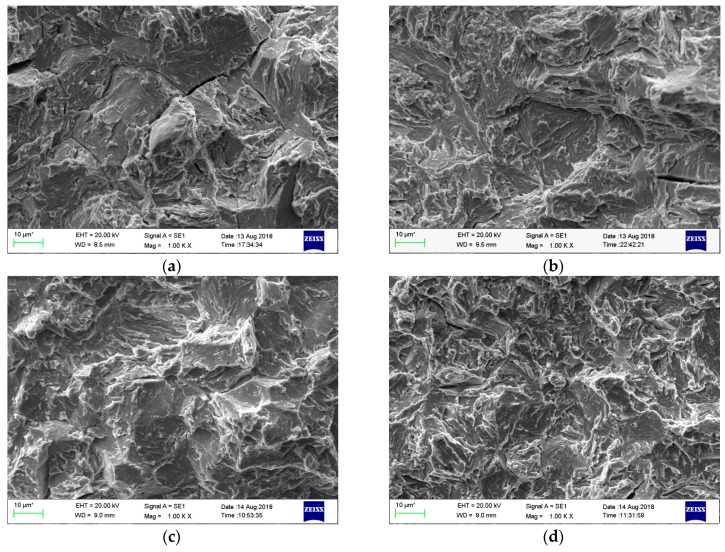
SEM fractographs in crack initiation region (the triangular area) of the hydrogen-charged specimens after SSRT: (**a**) DQ; (**b**) DQ-T100; (**c**) HS; (**d**) HS-T200.

Figure 11 shows the SEM micrographs in the crack initiation area (the triangular area) of all the hydrogen-charged SSRT specimens. The DQ specimen showed a mixed transgranular-intergranular fracture with some secondary cracks (Figure 11a), while all the other three specimens exhibited a predominant quasi-cleavage transgranular fracture accompanied by a few secondary cracks (Figure 11b,c,d). This result is generally in accordance with that of the SSRT tests. The tendency of intergranular fracture reveals that the prior austenite grain boundaries of the DQ specimen were severely influenced by the enrichment of hydrogen, while the crack initiation stress and/or the cohesiveness and energy required to grow the cleavage crack must have been lowered by hydrogen for the DQ-T100, HS and HS-T200 specimens.

## 4. Discussion

### 4.1. Influence of Tempering Treatment

A number of experiments have been done regarding the effect of tempering treatment on the HE behavior of high strength martensitic steels, and it is regarded that the HE susceptibility of this type of steels could be lowered through the increase in tempering temperature on the condition that temper embrittlement is avoided [24,33,34,35,36,37,38]. This improvement was mainly correlated with microstructural evolution, including the decrease in dislocation density, the formation and morphology variation of carbides as well as the unavoidable strength loss for traditional low-alloy martensitic steels when the tempering temperature was high [24,34,36]. As all the tested specimens exhibited an nearly identical strength level, it is thus rational to suggest that microstructural evolution rather than strength controls their HE susceptibility.

As shown in Figure 5 and Figure 7 and Table 1, there are a comparatively high amount of fine carbides for the DQ specimen although no tempering treatment was carried out. This result is mainly connected to the auto-tempering phenomenon of low-carbon steels, as the martensite-start temperature (*M*_s_) is ~421 °C for the tested low-carbon boron-alloyed steel (Figure 1), which is much higher than room temperature and can easy give rise to auto-tempering. Thus, its HE susceptibility is comparatively lower compared with that of medium-carbon martensitic steel with a similar strength level [38]. For the DQ specimen, there was still partial segregation of carbon in the form of filmy carbide at prior austenite grain boundaries, which would induce the segregation of hydrogen at highly stressed boundaries ahead of the notch tip [39], and thus caused part of the grain boundaries to embrittle and a low-stress intergranular fracture (Figure 11a).

There is a notable decrease of the *C*_Hr_ and a slight decrease of the *C*_H__i_ when the DQ specimen was tempered at 100 °C, while there are a notable decrease of the *C*_Hr_ and an increase of the *C*_H__i_ when the HS specimen was tempered at 200 °C (Figure 8). This phenomenon is mainly due to the decrease of dislocation density and internal stress (which is regarded as diffusible hydrogen trapping sites) as well as the increase of the amount of carbides (which is usually called irreversible hydrogen trapping site) [40,41]. The former could cause a decrease of hydrogen while the latter could cause an increase of hydrogen absorbed during hydrogen charging. The decrease of dislocation density and internal stress is more significant than the increase of carbide when the tempering temperature is low. As a result, the *C*_Hr_ of both the DQ-T100 and HS-T200 specimens notably decreased and the corresponding HE susceptibility was alleviated after low-temperature tempering. Moreover, the decrement of vacancies and occupation of hydrogen trapping sites around dislocation by interfacial solute atoms such as carbon due to low-temperature heat treatment was also responsible for the decrease of *C*_Hr_ [42]. By the way, as the difference of the amount of carbides between the DQ and DQ-T100 specimens is rather small, there was only a slight variation of the *C*_H__i_ between them, whereas a significant increase of the amount of carbides caused a notable increase of *C*_H__i_ when the HS specimen was tempered at 200 °C. Tempering treatment at 100 °C of the DQ specimen could notably lower its HEI value by a relative decrease of ~24% at no expanse of strength and ductility, which suggests that only auto-tempering is not enough while low-temperature tempering is still needed to further alleviate its HE susceptibility. After the tempering treatment, the increase of the fine carbides precipitated uniformly within grains caused the decrease in the tendency for intergranular fracture (Figure 11b,d), because most of the hydrogen was trapped within grains, as was also found by Nagao et al. in low-carbon martensitic plate steels [43].

### 4.2. Influence of Hot Stamping

**Figure 12:**
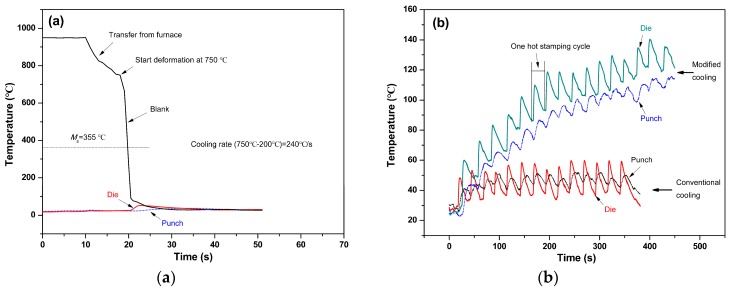
(**a**) Temperature evolution of blank and tools during the designed HS process of the tested steel and (**b**) temperature evolution of tools during continuous HS process.

As shown in Table 1, the HS specimen has comparable tensile properties in terms of strength and ductility to the DQ specimen; it exhibits much lower HE susceptibility than the latter, although its *C*_Hr_ is higher than that of the latter, which can be explained as follows. As mentioned above, both the microstructural characteristics and mechanical properties of the HS specimen are similar to those of the DQ-T100 specimen as well as the value of HEI. Moreover, the HEI value of the HS specimen is only slightly higher than that of the HS-T200 specimen. Therefore, it is reasonable to suppose that the HS specimen had experienced a low-temperature tempering during the HS process in addition to auto-tempering. To confirm this assumption, the temperature evolutions of the blank, punch and die during the HS process were detected in detail (Figure 12). Under the condition of using controlled water as coolant, the temperature of the die and punch during forming rose to about 60 °C and 50 °C after a single HS cycle, respectively (Figure 12a). The estimated cooling rate of the blank was ~240 °C/s, which is much higher than the critical cooling rate of getting full martensite for the tested steel. As shown in Figure 12b for the continuous HS practice, the temperature of the punch and die rose to ~50–60 °C and almost remained at that temperature even up to 14 HS cycles using the conventional cooling schedule, while it increased to ~120–130 °C after about 14 HS cycles and tended to remain at that temperature using the modified cooling schedule, which includes controlling of the temperature of coolant, the blank forming pressure and holding time. Similar results were also found by other researchers [3,8]. Since the tested HS specimens were cut from the blank pieces after 20 HS cycles during continuous HS process using the modified cooling schedule, the HS blank studied should have experienced a low-temperature tempering treatment.

From above considerations, it is thus proposed that the modified cooling schedule could be applied to practical HS process to alleviate the HE susceptibility of the tested low-carbon boron-alloyed steel, although thorough study is still needed in the future. By the way, automotive components are usually heated to around 170 °C during the paint baking process after forming, i.e., bake-hardening treatment [42,44]; this treatment not only slightly affects the mechanical properties, but also could be used to further alleviate the HE susceptibility of the hot stamped parts, as was confirmed by the results of the HS-T200 specimen.

It is known that stress including applied stress and residual stress besides hydrogen concentration, and material characteristics, such as strength level and microstructure, also play a significant role in affecting the HE behavior of high strength steels [15]. Therefore, it is worth noting that although the HE susceptibility of the HS specimen is still a little higher, the almost completely absence of residual tensile stress originating from the HS process can be beneficial to its HE susceptibility [14], compared with conventional automotive parts manufactured by the cold forming process of high strength martensitic steels, which usually causes high residual stresses because of the spring-back effect [2,3,4].

## 5. Conclusions

1. The designed HS schedule could obtain a fully fine martensitic microstructure with many fine dispersed carbides, which exhibited comparable mechanical properties to the DQ specimen. Low-temperature tempering treatment of the DQ and HS specimens gave rise to an increase of YS and TEL while a slight decrease of UTS occurred.
2. TDS analysis revealed that hydrogen-charging caused an additional remarkable hydrogen desorption peak at round 65–80 °C for both the DQ and HS specimens. The diffusible hydrogen concentration (*C*_Hr_) of the HS specimen is higher than that of the DQ specimen, and *C*_Hr_ was lowered after low-temperature tempering.
3. The DQ specimen exhibited a comparatively high HE susceptibility with HEI value of ~36%, while tempering treatment at 100 °C could notably alleviate it by a relative decrease of ~24% at no expanse of strength and ductility.
4. The HE susceptibility was significantly lowered for the HS specimen compared with that of the DQ specimen, and it is almost identical to that of the DQ-T100 specimen, which is mainly ascribed to the low-temperature tempering treatment around 120 °C during continuous HS process using the modified HS cooling schedule. Tempering at 200 °C could further alleviate the HE susceptibility of the HS specimen.
5. The DQ specimen showed a mixed transgranular-intergranular fracture, while all the other three specimens exhibited a predominant quasi-cleavage transgranular fracture accompanied by few secondary cracks.

## Figures and Tables

**Table 1:** Summary of tensile properties and microstructural characteristics of the samples under different conditions.

Sample	Condition	UTS (MPa)	YS (MPa)	TEL (%)	Mass Fraction of Carbide (%)	Dislocation Density (cm^−2^)
DQ	Quenched	1475	1120	12.0	0.075	7.90 × 10^11^
DQ-T100	Quenched + 100 °C × 2 h	1460	1165	12.5	0.082	-
HS	Hot stamped	1485	1170	12.0	0.103	1.13 × 10^12^
HS-T200	Hot stamped + 200 °C × 2 h	1455	1205	12.5	-	9.87 × 10^11^

**Table 2:** The SSRT results of the samples under different conditions.

Sample	UTS (MPa)	*σ*_N0_ (MPa)	*σ*_NH_ (MPa)	HEI (%)
DQ	1475	1817	1162	36.0
DQ-T100	1460	1817	1237	31.9
HS	1485	1819	1343	26.2
HS-T200	1455	1825	1383	24.2
